# Profiles of intuitive eating in adults: the role of self-esteem, interoceptive awareness, and motivation for healthy eating

**DOI:** 10.1186/s12888-024-05722-2

**Published:** 2024-04-17

**Authors:** Nancy Chammas, Anna Brytek-Matera, Debora Tornquist, Felipe Barreto Schuch, Zeinab Bitar, Diana Malaeb, Mirna Fawaz, Feten Fekih-Romdhane, Souheil Hallit, Sahar Obeid, Michel Soufia

**Affiliations:** 1 School of Medicine and Medical Sciences, Holy Spirit University of Kaslik, Jounieh, P.O. Box 446, Lebanon; 2 Eating Behavior Laboratory (EAT Lab), Institute of Psychology, University of Wrocław, 50-527 Wrocław, Poland; 3 Department of Sports Methods and Techniques, Federal University of Santa Maria, Santa Maria, Brazil; 4 Institute of Psychiatry, Federal University of Rio de Janeiro, Rio de Janeiro, Brazil; 5 Institute of Health Sciences, Universidad Autónoma de Chile, Providência, Chile; 6 Univ Rennes, Inserm, EHESP, Irset (Institut de recherche en santé environnement et travail)–, UMR_S 1085, F-35000 Rennes, France; 7 College of Pharmacy, Gulf Medical University, Ajman, United Arab Emirates; 8 College of Health Sciences, American University of the Middle East, Kuwait, Kuwait; 9 The Tunisian Center of Early Intervention in Psychosis, Department of Psychiatry “Ibn Omrane”, Razi hospital, 2010 Manouba, Tunisia; 10 Faculty of Medicine of Tunis, Tunis El Manar University, Tunis, Tunisia; 11 Applied Science Research Center, Applied Science Private University, Amman, Jordan; 12 Social and Education Sciences Department, School of Arts and Sciences, Lebanese American University, Jbeil, Lebanon

**Keywords:** Intuitive eating, Self-esteem, Interoceptive awareness, Healthy eating, Latent profile analysis

## Abstract

**Objective:**

Intuitive eating is an eating behavior that has recently come to use mainly in the young population. Knowing that the Lebanese cultural diet differs from other countries, the purpose of this study was to investigate if there is a relationship between self-esteem, interoceptive awareness, and motivation for healthy eating in a sample of Lebanese adults using a Latent Profile Analysis approach.

**Design:**

Cross-sectional study.

**Setting:**

Lebanese governorates.

**Participants:**

359 Lebanese participants enrolled in this study (mean age: 22.75 ± 7.04 years, 40.1% males), through convenience sampling in several Lebanese governorates. Participants were asked to fill anonymously the following scales: The Intuitive Eating Scale (IES-2), the Rosenberg Self-Esteem Scale, the Multidimensional Assessment of Interoceptive Awareness Scale (MAIA), and the Motivation for Healthy Eating Scale (MHES).

**Results:**

Our findings revealed four profiles: profile 1 (*n* = 67; 18.66%) characterized by high SE and intermediate interoceptive awareness and motivation for healthy eating; profile 2 (*n* = 86; 23.97%) presented high SE, interoceptive awareness, and motivation for healthy eating; profile 3 (*n* = 86; 23.96%) characterized by high SE, interoceptive awareness, and motivation for healthy eating; class 4 (*n* = 108; 30.08) described by low SE, intermediate interoceptive awareness, and motivation for healthy eating One-way analysis of variance did not observe a significant difference between the four profiles based on intuitive eating (F = 1.810; *p* = 0.145; ɳp2 = 0.015).

**Conclusions:**

Among a sample of Lebanese people, four profiles of interoceptive awareness, motivation for healthy eating, and self-esteem were observed, with no difference concerning intuitive eating.

## Introduction

Intuitive eating (IE) is an evidence-based health approach that has been on the rise over recent years, particularly among young adults. It emphasizes the importance of relying on one’s body to guide food choices, free from the influence of diet culture or judgment. Simply put, IE is about trusting our body’s natural hunger and satiety signals to make informed dietary decisions. Hence, it focuses on a person’s physiological internal hunger and satiety to regulate their eating habits [1]. Contrary to restrictive diets, which can cause anxiety [2], eating for physical, rather than emotional, reasons helps regulate emotions. By relying on internal hunger and satiety cues, individuals can determine the timing and amount of food needed. This, in turn, leads to better psychological well-being and congruence between the body and food choices [3].

There is a positive relationship between one’s own body and their food choice, since IE emphasizes self-trust and promotes eating in accordance with satiety and hunger signs [4]. Thus, individuals practicing IE develop a sense of empowerment and confidence in their ability to nourish and care for their bodies, ultimately promoting self-esteem and a healthier relationship with themselves [5].

Self-Esteem (SE) is a global evaluation of one’s self-worth [6], and its association with body image is widely investigated [7, 8]. Furthermore, it has been linked to producing several psychological benefits and high levels of positive affect, by enabling the individual to maintain a favorable attitude and confidence about oneself. Cash and Fleming [9] reported that women who are dissatisfied with their appearance, body shape and size have a low SE. In contrast, women who have a high SE are more likely to have a positive evaluation of their body [7]. For instance, choosing food according to internal feelings will give the person a sense of mastery, fostering confidence while connecting with internal cues. Overall, building one’s SE may be a helpful strategy for individuals who seek to improve their relationship with food and adopt a more intuitive approach to eating.

Furthermore, individuals should be able to recognize and understand signals coming from within the body to successfully adopt IE. Interoceptively aware people frequently make decisions that promote their physical and mental wellbeing, because they are more aware of their bodies’ demands [10], and have healthier relationships with food and less disordered eating behaviors [11]. For example, sensitivity for interoceptive processes may be deficient in anorexia nervosa (AN) and an inability to perceive and process bodily signals adequately has been considered as a predictor and maintaining factor of AN [12]. In the same line of thought, interoceptive awareness is a key component of IE, enabling individuals to attune to their body and make informed decisions about their food choices. This involves recognizing and comprehending signals of fullness, hunger, and other sensations like heart rate, breathing, and muscle tension [13]. Improvements in interoceptive awareness have shown positive effects on mental health by encouraging self-awareness and self-regulation. Accordingly, by tuning into their body’s signals and understanding what they mean, the person can make more informed decisions about what and when to eat [13].

In addition, IE influences an individual’s ability to maintain healthy habits. To adopt and maintain the IE lifestyle, motivation is the key; it can be a powerful tool to inspire healthier eating habits, and is an important factor in developing the necessary skills to practice IE [14]. In the context of motivation, both internal and external factors can act as motivators [15]. The benefits of eating a healthy, balanced diet include lowering the risk of developing chronic diseases (like diabetes and heart disease), boosting energy, promoting vitality, as well as improving the mental health of the individual by reducing the symptoms of anxiety and depression [16]. A person who is motivated to eat healthy is more likely to choose foods that are high in nutrients, providing both nourishment and energy [14]. When choosing healthy foods that meet their bodies’ needs, individuals can become more aware of their hunger and fullness cues, make proper food choices, and attend to their physical and emotional needs.

IE is a new eating behavior emerging specifically within the Lebanese population. To our knowledge, there are no studies yet assessing the relationship of SE, interoceptive awareness, and motivation for healthy eating with IE. As far as we know, Lebanese families and friends have a tradition of gathering frequently to eat together, considering meals as a way to communicate. These habits are common in the Eastern region [17]. In contrast, Western cultures encourage the individual follow a strict diet to lose weight, aiming to boost self-confidence [18]. In other words, people who have high SE may be more naturally inclined to adopt healthy lifestyle choices, such eating a balanced diet, which supports their overall wellness [5]. Self-satisfied individuals are more likely to have confidence in their ability to overcome obstacles, which helps maintain motivation for a healthy diet. In this study, as the Lebanese diet differs from that of other countries, it is important to recruit participants from different governorates to reflect the diversity and enhance external validity. Each governorate has its own socioeconomic factors, dietary habits, lifestyle pattern and culture. Consequently, the purpose of this study was to examine the relationship between IE, SE, interoceptive awareness and motivation for healthy eating among a sample of Lebanese adults using a Latent Profile Analysis approach.

## Methods

### Ethics approval and consent to participate

The Ethics and Research Committee at the Lebanese International University approved this study protocol (2022RC-051-LIUSOP). A written informed consent was considered obtained from each participant when submitting the online form.

### Study design

A total of 359 Lebanese participants were enrolled in this cross-sectional study that was conducted between September and November 2022, through convenience sampling in several Lebanese governorates. The survey was a Google form questionnaire that was administered through the internet, using the snowball technique. Participants were informed about the study, and were provided an online link to it; pressing on the link led interested participants to the consent form and information form (outlining the current study’s objectives, anonymity, and voluntary permission to research). When confidentiality is assured, participants are encouraged to respond honestly and deliver more accurate information. Secondly, detailed instructions defining the purpose of the survey and the importance of the thoughtfulness of the responses minimized inaccuracy.

### Minimal sample size calculation

According to a previous research [19], a sample size of at least 300 subjects is suggested for conducting Latent Class Analyses.

### Measures

The questionnaire used was anonymous and in Arabic, the native language in Lebanon. It required approximately 10 to 15 min to complete. It consisted of three parts. The first part explained the study’s topic and objective, a statement ensuring the anonymity of respondents. The participant had to select the option stating *I consent to participate in this study* to be directed to the questionnaire.

The second part gathered participants’ sociodemographic information, including age and sex. The Household Crowding Index (HCI), a measure of family’s socioeconomic status [20], was calculated by dividing the number of individuals living in the house by the number of rooms (excluding the kitchen and the bathrooms) [21]. The heights and weights of participants were self-reported to calculate the Body Mass Index (BMI) (kg/m2).

The 23 items on the *Intuitive Eating Scale (IES-2)* add up to an overall IE score that is calculated by summing up all the items and dividing the total by 23. This scale has four subscales: Body-Food Choice Congruence (B-FCC) with three items (scored by adding 21 to 23 and dividing by 3), Eating for Physical Rather than Emotional Reasons (EPR) with six items (scored by adding 7 to 14 and dividing by 8), Unconditional Permission to Eat (UPE) with six items (scored by adding 1 to 6 and dividing by 6), Reliance on Hunger and Satiety Cues (RHSC) with eight items (scored by adding 15 to 20 and dividing by 6) [22]. The response options ranged from 1 (strongly disagree) to 5 (strongly agree). The higher the scores, the higher the IE. The validated Arabic version has been used [23] (α = 0.84).

The *Rosenberg Self-Esteem Scale* is a 10-item scale that measures both positive and negative feelings about the self [24]. Response choices on the scale, which is regarded as unidimensional, range from “strongly agree” to “strongly disagree”. One point is given for “Strongly Disagree”, two points are given for “Disagree”, three points are given for “Agree”, and four points are given for “Strongly Agree”. The ratings for each of the ten items are then added together to create a continuous scale. Higher SE levels are indicated by higher scores on this scale. In this study, we used the validated Arabic version of this scale [25] (α = 0.81).

*The Multidimensional Assessment of Interoceptive Awareness (MAIA-2)*: Validated in Arabic [26], is suitable for adults (18 + years), and has 37 items. The MAIA consists of 8 subscales: “Noticing” with 4 items (scored by adding 1 t 4 and dividing by 4), “Not-Distracting” with 6 items (scored by adding items 5 to 10 then dividing by 6), “Not-Worrying” with 5 items (scored by adding 11 to 15 then dividing by 5), “Attention Regulation” with 7 items (scored by adding 16 to 22 then dividing by 7), “Emotional Awareness” with 5 items (scored by adding 23 to 27 then dividing by 5), “Self-Regulation” with 4 items (scored by adding 28 to 31 then dividing by 4), “Body Listening” with 3 items (scored by adding items 32 to 34 then divide by 3), “Trusting” with 3 items (scored by adding items 35 to 37 then divide by 3) [27]. The response options ranged from 0 to 5, where 0 corresponds to never and 5 to always on a six-point Likert scale, with higher score equates to more awareness of bodily sensation. The Cronbach’s alpha values for the subscales were as follows: notice (α = 0.86), not distracting (α = 0.89), not worrying (α = 0.81), attention regulation (α = 0.93), emotional awareness (α = 0.90), self-regulation (α = 0.90), body listening (α = 0.88) and trust (α = 0.90).

*The Motivation for Healthy Eating Scale (MHES)* includes 31 items and consists of 6 subscales: intrinsic motivation with 5 items, integrated motivation with 5 items, identified regulation with 5 items, introjected regulation with 5 items, external regulation with 6 items, amotivation with 5 items [15]. Each item was rated on a 7-point scale, ranging from 1 (Does not correspond at all) to 7 (Corresponds very well), with higher scores indicating higher motivation to healthy eating. The Cronbach’s alpha values for the subscales were as follows: intrinsic motivation (α = 0.78), integrated regulation (α = 0.83), identified regulation (α = 0.87), introjected regulation (α = 0.82), external regulation (α = 0.85) and amotivation (α = 0.79). The forward and backward translation method was applied; the English version was translated to Arabic by a Lebanese translator who was completely unrelated to the study. Afterwards, a Lebanese psychologist with a full working proficiency in English, translated the Arabic version back to English. The initial English version and the second English version were compared to detect and later eliminate any inconsistencies. A pilot study was conducted on 20 persons before the start of the official data collection to make sure all questions are well understood; no changes were done consequently.

### Statistical analysis

First, To check the psychometric properties of the MHES, we employed an EFA-to-CFA [28] strategy using the RStudio (Version 1.4.1103 for Macintosh) the Lavaan and semTools packages. The original sample was randomly divided into two subsamples, one used for the EFA (*n* = 131) and the other for the CFA (*n* = 228). The Kaiser-Meyer- Olkin (KMO) measure of sampling adequacy, and Bartlett’s test of sphericity was used to ensure the adequacy of the data [29]. In EFA, the number of factors underlying MHES items was determined on the basis of the screen test [30]. Based on the factors from the EFA, we conducted a Confirmatory Factor Analysis (CFA) using the data from the second subsample. Parameter estimates were obtained using the following fit indices: Comparative Fit Index (CFI), the Tucker-Lewis Index (TLI), Goodness of Fit Index (GFI), Standardized Root Mean Square Residual (SRMR) and Root Mean Square Error of Approximation (RMSEA). Values ≥ 0.90 for the GFI, CFI and TLI indicate a good model fit, values at or below 0.08 for the RMSEA, and 0.06 for the SRMR indicate good fit of the model to the data [31].

Afterwards, we transformed the self-esteem, interoceptive awareness, and motivation for healthy eating scales using the Percentage of Maximum Possible (POMP) method [32]. The POMP transformation makes each scale range from 0 to 100 while maintaining the proportions of the differences between the observed values. Subsequently, we used Latent Profile Analysis (LPA), a latent class analysis with continuous data [33], to identify subgroups based on self-esteem, interoceptive awareness, and motivation for healthy eating in the sample and including the covariates age, sex, body mass index, household crowding index, physical activity, and financial burden. Models with up to five latent profiles were tested, considering that the LPA method does not determine the number of classes a priori. The different models were examined, and compared regarding statistical criteria, sample size included in the profiles, and interpretability of the profile [33]. Among the statistical criteria, we evaluated the fit of the models using two criteria: Akaike Information Criterion (AIC) and Bayesian Information Criterion (BIC), where lower values indicate better models. For LPA we used the Stata software, version 15.

Profiles’ differences regarding SE, interoceptive awareness, and motivation for healthy eating levels were tested using one-way ANOVA (Analysis of Variance). Bonferroni post hoc tests were conducted to the groups two by two. Partial eta squared (ɳ_p_2; representing the effect size) was also recorded. ɳ_p_2 values of 0.01, 0.06 and 0.14 indicate small, medium and large effects respectively [34]. *P* < 0.05 was considered significant. No missing values were found since all questions were required [35]. Cronbach’s alpha values were conducted to assess scales’ reliability. The skewness ( = − 0.428) and kurtosis (= 0.052) of the IE score was considered normally distributed since they varied between − 1 and + 1 [36]. The SPSS software version 23 (IBM Corp., Armonk, NY, USA) was used to conduct this analysis.

## Results

A total of 359 participants enrolled in this study (mean age: 22.75 ± 7.04 years, 40.1% males). Other sociodemographic characteristics are summarized in Table 1.

**Table 1:** Sociodemographic and other characteristics of the participants

Variable	n (%)
Sex	
Male	144 (40.1%)
Female	215 (59.9%)
	**Mean ± SD**
Age, years	22.75 ± 7.04
Body Mass Index (kg/m^2^)	24.12 ± 5.13
Intuitive eating	43.14 ± 0.36
Self-esteem	28.44 ± 5.49
Interoceptive awareness	
Noticing	8.82 ± 3.83
Not distracting	13.59 ± 6.50
Not worrying	10.63 ± 5.07
Attention regulation	16.13 ± 7.74
Emotional awareness	12.51 ± 6.04
Self-regulation	9.10 ± 4.68
Body listening	6.45 ± 3.51
Trust	7.47 ± 3.81

### Exploratory factor analysis

The data viability for factorability was evaluated through Kaiser–Meyer–Olkin (KMO) measure of sample adequacy was 0.91 and Bartlett’s test of Sphericity (*P* < 0.05). (See Table 2)

**Table 2:** Factor loadings deriving from the exploratory factor analysis

MHES Items	F1	F2	F3
3	0.83		
5	0.87		
6			0.68
7	0.61		
8			0.78
10			0.65
11	0.86		
12	0.89		
15	0.86		
16		0.47	
17		0.62	
22		0.69	
23		0.86	
24		0.94	
25		0.54	
26		0.49	
28		0.58	

### Confirmatory factor analyses

CFA indicated that fit of the three-factor model obtained from the EFA was poor: χ^2^/df = 720.01/132 = 5.45, RMSEA = 0.014 (90% CI 0.013, 0.015), SRMR = 0.08, CFI = 0.86, TLI = 0.83. We added a correlation between items 23–24 due to high modification indices; the results improved as follows: χ^2^/df = 553.86/131 = 4.23, RMSEA = 0.012 (90% CI 0.011, 0.013), SRMR = 0.07, CFI = 0.90, TLI = 0.88.

### Identification and description of the profiles

Table 3 describes the criteria used to evaluate the fit of the models for the different profiles solutions generated by the LPA based on self-esteem, interoceptive awareness, and motivation for healthy eating. Increasing the number of profiles, resulted in steadily decreasing value of BIC and AIC. However, in the 5-group model, the additional group represented a low proportion of the sample, presenting fewer than 50 cases. Some LCA scholars argue that researchers should not consider classes with fewer than 50 cases [37]. Therefore, the four profiles model was more appropriated and adopted for the study.

**Table 3:** Statistical fit indices for different profiles solutions

Number of Profiles	Smallest group %*	AIC	BIC
1	100.00	59384.71	59617.71
2	46.77	57797.65	58189.86
3	28.15	57221.28	57772.72
4	18.27	56832.77	57543.42
5	12.80	56564.08	57433.94

AIC = Akaike Information criteria

BIC = Bayesian information criterion

*Estimation of the proportion of the sample assigned to the smallest group

**Fig. 1:**
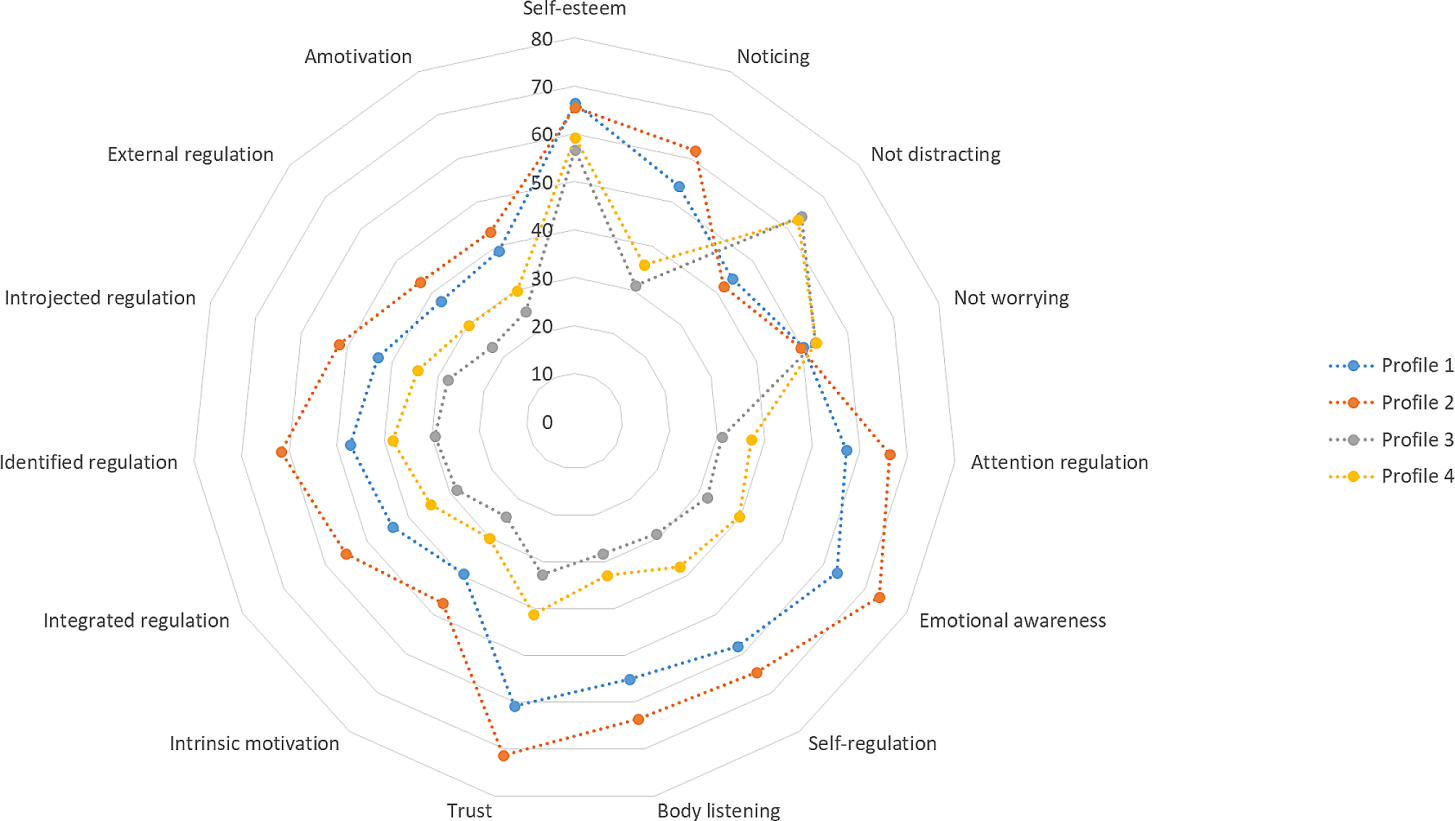
Final latent profiles for self-esteem, interoceptive awareness, and motivation for healthy eating

Table 4 presents the estimated mean of each variable for each profile, and describes the characteristics of each group for the four-profile model. The final latent profiles are presented in Fig. 1. The first profile (*n* = 67; 18.66%) was characterized by high SE and intermediate interoceptive awareness and motivation for healthy eating. The second group (*n* = 86; 23.96%) showed high SE, interoceptive awareness, and motivation for healthy eating. A third group (*n* = 98; 27.30%) was characterized by low SE, interoceptive awareness, and motivation for healthy eating. The fourth profile (*n* = 108; 30.08) was described as having low SE, intermediate interoceptive awareness, and motivation for healthy eating.

**Table 4:** Estimated mean for each item in each profile (n = 359)

	Profile 1*	Profile 2*	Profile 3*	Profile 4*
Self-esteem	66.50	65.53	56.69	59.12
Noticing	53.67	61.86	31.09	35.83
Not distracting	44.43	42.04	63.79	62.74
Not worrying	50.31	49.65	52.91	53.05
Attention regulation	57.09	66.20	30.89	37.18
Emotional awareness	63.39	73.56	32.02	39.74
Self-regulation	58.07	64.79	28.99	37.47
Body listening	55.08	63.55	28.20	32.94
Trust	60.74	71.39	32.78	41.29
Intrinsic motivation	39.45	46.98	24.50	30.26
Integrated regulation	44.01	55.20	28.48	34.75
Identified regulation	47.10	61.62	29.36	38.25
Introjected regulation	43.24	51.67	27.83	34.44
External regulation	37.53	43.30	23.12	29.85
Amotivation	38.86	43.30	25.13	29.75

*Means of scales transformed by the Percentage of Maximum Possible (POMP) method

### Comparison between the four profiles

Using one-way analysis of variance and the Bonferroni post-hoc tests (Table 5), we compared the four profiles based on IE. No significant difference was observed between the four profiles described (F = 1.810; *p* = 0.145; ɳp2 = 0.015).

**Table 5:** Typology of individuals based on self-esteem, interoceptive awareness and motivation for healthy eating and its association with intuitive eating

Profile 1 (n = 67)*	Profile 2 (n = 86)*	Profile 3 (n = 98)*	Profile 4 (n = 108)*	F	*p*	ɳ_p_2	
43.19 ± 3.90	43.90 ± 4.01	42.61 ± 3.47	42.99 ± 3.90	1.810	0.145	0.015	

*Numbers displayed as mean ± standard deviation of IE scores

## Discussion

IE, which emphasizes using body language and intuition to guide eating patterns, promotes a positive relationship with food. This study showed that different profiles of SE, interoceptive awareness, and motivation for healthy eating do not differ concerning IE behavior, within the cultural and contextual complexity of the Lebanese culture. By using Latent Profile Analysis, different profiles or groups of people with comparable combinations of behavioral and psychological qualities can be found.

Profile 3 was characterized by low SE, interoceptive awareness and motivation for healthy eating, while profile 2 antagonizes with profile one, being characterized by high SE, high interoceptive awareness but low moderate healthy eating motivation. A very high SE is associated with high IE in previous studies [38, 39]. For example, a person is less likely to engage in restricted eating habits or disordered eating behaviors when they have greater SE [40] because they are more likely to have a positive attitude toward their body. Additionally, they are inclined to trust their body’s intrinsic signals for hunger and fullness, basing their eating decisions on those requirements rather than on external influences or emotional responses (like societal beauty standards) [41]. Hence, they are more likely to prioritize themselves and engage in self-care activities like IE. The basis of self-respect is the link between IE and strong SE [43]. People who feel positive about themselves are more likely to treat their bodies with respect while they eat. Their decisions are based on internal cues rather than society norms or external influences, and they are aware of and accept their hunger signals [42]. This adherence to IE principles represents a healthy relationship with food, unbound by the judgments associated with diet culture and its negative impact on mental and physical well-being [43]. Furthermore, SE and self-confidence are intertwined with a person’s sense of autonomy; they believe they can make choices that are good for them and trust that their bodies will guide them toward a balanced and satisfying relationship with food [44]. In this way, they confront diet culture and social conventions that have the potential to damage the sense of self-worth and prioritize their own wellbeing and values over those of others. In addition, individuals who are more likely to employ healthier methods of emotion management and reduce their reliance on food as a primary coping mechanism tend to have a stronger sense of self-worth [45], and greater emotional resilience. They are more capable of using healthy coping mechanisms to manage stress, anxiety, and other emotional difficulties. Instead of resorting to unhealthy coping strategies, individuals could take part in ways to promote emotional well-being [46]. Hence, supporting IE involves enabling people to understand and respond to their emotional needs without relying solely on food.

Profile 1 was characterized by high SE, and intermediate interoceptive awareness and motivation for healthy eating. In contrast, profile 4 was characterized by low SE, and intermediate interoceptive awareness and motivation for healthy eating. The term ‘motivation’ refers to internal or external factors that affect people to make dietary choices that improve their physical and emotional well-being. Personal beliefs, dietary expertise, a desire for physical well-being, or external constraints like society conventions or professional medical advice can all contribute to this motivation [15]. People with intrinsic motivation are said to produce better results, have higher levels of interest and confidence, and experience better overall health outcomes, including improved metabolic health, weight maintenance, and body satisfaction, in comparison to those who are externally controlled for an action [15]. As demonstrated in the present study, profile 1 englobed a positive SE and moderate levels of interoceptive awareness. Individuals with a certain level of SE may perceive their abilities’ areas for improvement in a clear and consistent manner [38]. People’s SE may rise if they succeed in making healthy food choices, which may, in turn, support healthy behaviors in a positive feedback loop [47]. An optimistic and body-accepting mindset is fostered by IE. People who have a positive body image and SE are more likely to follow IE guidelines [41], since positivity about oneself may make the individual less vulnerable to social influences and less likely to follow externally imposed dieting rules [48]. Finally, people who have a moderate level of awareness are neither overly concentrated nor totally devoid of their body’s messages [49]. They are more capable of controlling how they respond when they are hungry or full, and how to stay out of extremes and balance their eating habits by paying close attention to the subtle cues their bodies provide them [50]. Individuals with high SE feel more comfortable with themselves and their beliefs [7]. They are more attuned to their internal cues, leading to greater motivation to maintain a healthy relationship with food.

Moreover, individuals with interoceptive awareness can identify and correctly interpret the signs of hunger and fullness emanating from their bodies. In this context, higher interoceptive awareness makes people more sensitive to the bodily sensations, facilitating the discrimination between physiological hunger and other environmental or emotional signals that induce eating. By respecting their appetite, eating only when they are actually hungry, and ending when they are comfortably full, people may eat more intuitively [10]. Many studies showed that IE and interoceptive awareness are associated [51, 52]; but since the current study used cross-sectional data, it is impossible to infer causal directions. Furthermore, body acceptance and appreciation are directly related to interoceptive awareness [53]. Individuals can create a closer relationship with their bodies and adopt a more supportive attitude toward them by tuning into internal physical sensations. This appreciation and acceptance of their bodies encourages a compassionate, nonjudgmental approach to food selection and eating experiences, promoting IE [54]. It is important to stress out that interoceptive awareness is frequently disrupted in people with eating disorders [55]. They might struggle to accurately perceive and interpret internal sensations, such as craving, satiety, and fullness. For instance, individuals with AN may disregard or misjudge their body’s signs of hunger, leading to extreme limitation of food consumption, and resulting in lower IE behavior [52].

Individuals with low SE may find it difficult to trust, and pay attention to, their own body’s signals and requirements. They struggle to connect with their bodies and interpret internal cues like hunger and fullness, which could lead to feeling less confident and unsatisfied with their bodies [56]. Due to this mismatch, people may rely too much on external variables, such as rigid dietary regulations or cultural expectations, as opposed to respecting their own physical cues. A detachment from natural cues, resulting in irregular eating patterns and potentially dangerous food choices, might be attributed to this impaired interoceptive awareness [12]. This lack of drive frequently manifests as emotional eating or dependence on comfort foods as a coping method, perpetuating a cycle of declining SE [57]. In addition, people with poor SE may use food as a coping mechanism for unpleasant feelings, seeking comfort or a diversion from their emotions [58].

### Clinical implications

Psychiatrists and psychologists may consider it a priority to address interoceptive awareness concerns and SE problems as part of the treatment plan when dealing with patients who have eating disorders or disordered eating behaviors [12]. This could mean adopting therapies that promote body positivity, self-acceptance, and self-compassion, in addition to techniques that improve interoceptive awareness, such as mindfulness exercises.

Furthermore, the establishment of various profiles based on the variables emphasizes the significance of tailored treatment strategies. Clinicians can utilize this data to customize treatments or objectives depending on each patient alone. For example, people in profile 1 could gain from improving their interoceptive awareness skills and motivation for healthy eating, whereas those in profile 4 might require additional treatments to raise their poor SE and increase their enthusiasm for healthy eating. Therapy centered on self-acceptance, body image, and SE may be beneficial. This might entail employing cognitive-behavioral techniques to get rid of inaccurate self-perceptions, promote self-care behaviors, and develop a positive self-image.

### Limitations

This study was conducted during the economic crises that have affected the Lebanese community. The findings might not be applicable to the entire population because of a convenient sampling method used in this study. The research experienced data collection obstacles, such as information bias, which occurs when subjects struggle to recollect past events or details about their eating patterns. Another limitation is selection bias, as the snowball technique was used, with participants recruiting others from their social circles. In addition, the psychometric properties of the Arabic version of the MHES scale were not very good; therefore, our results should be interpreted with caution. Due to a lack of validation, this measure may be interpreted and understood differently, which might result in inaccurate data collected. Finally, because it is a cross-sectional research, it cannot assess variable variation over time or demonstrate causality; it only examines data collected from a sample at a specific point in time.

## Conclusion

In conclusion, four profiles of interoceptive awareness, motivation for healthy eating, and self-esteem were observed, with no difference concerning intuitive eating. In future perspectives, given IE’s rise to prominence, it would be interesting to study other characteristics associated with it, including its potential benefits for persons diagnosed with eating disorders.

## Data Availability

The datasets generated and/or analysed during the current study are not publicly available due to restrictions from the ethics committee but are available from the corresponding author on reasonable request.
